# Prospective Diagnostic Accuracy and Technical Feasibility of Artificial Intelligence-Assisted Rib Fracture Detection on Chest Radiographs: Observational Study

**DOI:** 10.2196/77965

**Published:** 2026-01-29

**Authors:** Shu-Tien Huang, Liong-Rung Liu, Ming-Feng Tsai, Ming-Yuan Huang, Hung-Wen Chiu

**Affiliations:** 1 Department of Emergency Medicine, Mackay Memorial Hospital, Taipei, Taiwan; 2 Graduate Institute of Biomedical Informatics, College of Medical Science and Technology, Taipei Medical University, 9F, Education & Research Building, Shuang-Ho Campus, No. 301, Yuantong Rd., Zhonghe Dist., New Taipei City, 235603, Taiwan, 886 266202589 ext 10929; 3 College of Medicine, Mackay Medical University, New Taipei City, Taiwan; 4 Division of Plastic Surgery, Mackay Memorial Hospital, Taipei, Taiwan; 5 Clinical Big Data Research, Taipei Medical University Hospital, Taipei City, Taiwan

**Keywords:** artificial intelligence, chest radiograph, clinical decision support, digital health, emergency radiology, feasibility study, faster R-CNN, real-world data, rib fracture

## Abstract

**Background:**

Rib fractures are present in 10%‐15% of thoracic trauma cases but are often missed on chest radiographs, delaying diagnosis and treatment. Artificial intelligence (AI) may improve detection and triage in emergency settings.

**Objective:**

This study aims to evaluate diagnostic accuracy, processing speed, and technical feasibility of an artificial intelligence–assisted rib fracture detection system using prospectively collected data within a real-world, high-volume emergency department workflow.

**Methods:**

We conducted an observational feasibility study with prospective data collection of a faster region-based convolutional neural network–based AI model deployed in the emergency department to analyze 23,251 real-world chest radiographs (22,946 anteroposterior; 305 oblique) from April 1 to July 2, 2023. This study was approved by the Institutional Review Board of MacKay Memorial Hospital (IRB No. 20MMHIS483e). AI operated passively, without influencing clinical decision-making. The reference standard was the final report issued by board-certified radiologists. A subset of discordant cases underwent post hoc computed tomography review for exploratory analysis.

**Results:**

AI achieved 74.5% sensitivity (95% CI 0.708-0.780), 93.3% specificity (95% CI 0.930-0.937), 24.2% positive predictive value, and 99.2% negative predictive value. Median inference time was 10.6 seconds versus 3.3 hours for radiologist reports (paired Wilcoxon signed-rank test *W*=112 987.5, *P*<.001). The analysis revealed peak imaging demand between 08:00 and 16:00 and Thursday-Saturday evenings. A 14-day graphics processing unit outage underscored the importance of infrastructure resilience.

**Conclusions:**

The AI system demonstrated strong technical feasibility for real-time rib fracture detection in a high-volume emergency department setting, with rapid inference and stable performance during prospective deployment. Although the system showed high negative predictive value, the observed false-positive and false-negative rates indicate that it should be considered a supportive screening tool rather than a stand-alone diagnostic solution or a replacement for clinical judgment. These findings support further clinician-in-the-loop studies to evaluate clinical feasibility, workflow integration, and impact on diagnostic decision-making. However, interpretation is limited by reliance on radiology reports as the reference standard and the system’s passive, non-interventional deployment.

## Introduction

Digital health technologies, particularly artificial intelligence (AI), are increasingly used to address diagnostic delays in high-acuity clinical settings. In emergency departments, timely identification of injuries is essential, yet radiographic interpretation remains constrained by heavy workloads and the inherent complexity of imaging—especially for subtle findings such as rib fractures.

Rib fractures are a frequent consequence of thoracic trauma, occurring in 10%‐15% of trauma patients and often indicating more serious underlying injuries [1-3]. When missed, they may lead to inadequate pain management, delayed respiratory support, pneumonia, or even preventable intensive care unit admissions. Beyond clinical harm, undetected fractures also carry medicolegal implications and increase health care costs.

Despite their significance, rib fractures are notoriously difficult to detect on chest radiographs (CXRs)—the first-line imaging modality in most emergency departments—due to overlapping anatomical structures and subtle fracture lines. Reported sensitivities for radiologist detection can be as low as 15%, with up to half of fractures potentially missed in high-volume settings [4,5]. Although computed tomography (CT) and ultrasound can improve accuracy, they are resource-intensive and not always feasible for frontline triage [6-8]. These limitations highlight an urgent need for AI-driven tools that can assist clinicians by rapidly identifying suspected rib fractures in routine CXRs, enabling more effective prioritization and timely intervention.

Recent advances in AI, particularly deep learning, have demonstrated strong potential in automating image analysis tasks across medical domains, including dermatology, ophthalmology, and pulmonary imaging [9-11]. Deep learning models, especially convolutional neural networks, can automatically extract complex image features and have shown superior performance compared to traditional machine learning methods in various image classification tasks [11-13]. Transfer learning further enables the adaptation of pretrained convolutional neural networks—originally developed for natural images—for medical image classification tasks, including bone fracture detection [14,15].

Although prior studies have applied deep learning to rib fracture detection with promising results, most were retrospective, limited in scale, and did not assess feasibility in operational emergency department workflows [7,16,17]. These proof-of-concept efforts did not address the practical barriers to integrating AI into emergency radiology workflows, such as inference latency, system interoperability, or artifact handling.

To address this gap, we conducted an observational feasibility study with prospective data collection, evaluating an AI model for rib-fracture detection on CXRs. The system was passively deployed in parallel with routine emergency department imaging workflows using real-world data, without influencing clinical decisions. This design allowed the assessment of diagnostic performance, processing speed, and operational characteristics within standard clinical workflows.

## Methods

### Study Design

The observational feasibility study protocol was reviewed and approved by the Institutional Review Board of MacKay Memorial Hospital (IRB No. 20MMHIS483e) prior to the initiation of data collection. MacKay Memorial Hospital is a tertiary referral and level 1 trauma center in northern Taiwan. The AI system functioned passively in real time without influencing clinical decisions or patient management. As the system functioned in a noninterventional, observational manner, prospective trial registration was not required.

**Figure 1.**
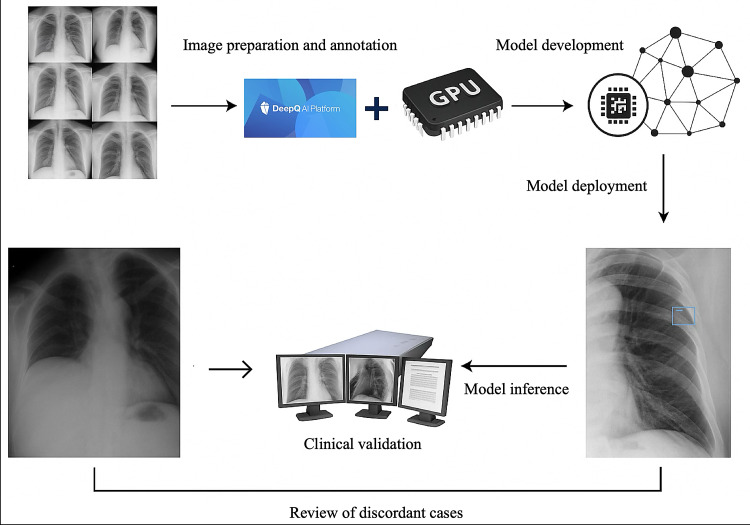
Study workflow of a prospective observational feasibility study evaluating an artificial intelligence–assisted rib fracture detection system using chest radiographs in patients admitted to the emergency department at a high-volume tertiary medical center (April 1-July 2, 2023). GPU: graphics processing unit.

From April 1 to July 2, 2023, all chest and rib radiographs acquired in the emergency department were automatically processed by the AI system in near-real-time. Both standard CXRs and rib-only views acquired during the study period were automatically analyzed, as both modalities are routinely used for suspected thoracic trauma. During the study period, a temporary 14-day graphics processing unit (GPU) hardware outage occurred, during which radiographs were not processed in real time; these examinations were excluded from turnaround time analysis but retained for diagnostic accuracy, as formal radiology reports were available. No additional exclusion criteria were applied beyond the 14-day system outage; all eligible emergency radiographs during the study period were included in the analysis. The system operated passively alongside routine clinical workflows, without influencing clinical decisions. AI-identified suspected rib fractures were highlighted using bounding boxes on a backend interface, which was accessible only for research evaluation and remained hidden from the clinical care team (Figure 1).

### Ethical Considerations

This study was approved by the Institutional Review Board of MacKay Memorial Hospital (IRB No. 20MMHIS483e) and conducted in accordance with the Declaration of Helsinki. Informed consent was waived because the study involved secondary analysis of routinely acquired, deidentified clinical imaging data, and the AI system operated passively without influencing patient management.

All data were deidentified prior to analysis and processed on secure institutional servers with access limited to authorized research personnel. No compensation was provided to participants. All images included in the manuscript were fully anonymized, and no identifiable patient information is disclosed.

Consistent with the approved observational study design, all AI outputs—including discordant cases—were withheld from treating clinicians and did not influence patient management.

### AI Model Development

CXRs were retrospectively collected from the hospital picture archiving and communication system (PACS) for model training. All images were deidentified and preprocessed using histogram equalization and image inversion to improve fracture conspicuity. Fracture locations were annotated using bounding boxes by a board-certified emergency physician with 18 years of clinical experience via the DeepQ AI platform [18].

A deep learning model was developed using PyTorch (v1.13) with GPU acceleration. The architecture was based on faster region-based convolutional neural network (R-CNN) [19], incorporating a ResNet-50 backbone for feature extraction, a region proposal network for candidate region generation, and a classification head for fracture detection.

The dataset comprised 2079 CXRs (1065 fracture-positive and 1014 normal) collected between 2010 and 2020. Images were randomly divided into training (80%) and validation (20%) sets at the image level, as each radiograph represented an independent study. When multiple images were obtained from the same patient encounter, each radiograph was treated as an independent sample. Data augmentation—including random rotation, flipping, brightness, and contrast adjustment—was applied to improve generalization. To address the inherent class imbalance given the low fracture prevalence, class-weighted loss and oversampling of fracture-positive images were employed.

### Model Validation

Model performance was assessed on a hold-out test set of 262 CXRs containing 724 expert-annotated rib fractures. Evaluation metrics were reported at both the case and object levels.

At the case level, the unit of analysis was the radiographic study. A study was considered positive if at least 1 rib fracture was detected, regardless of the number of fractures present. The model correctly identified fractures in 230 of 257 fracture-positive studies, achieving a sensitivity of 89.5%. With only 8 false-positive cases, precision reached 96.6%, yielding an overall *F*_1_-score of 0.93.

At the object level, performance reflected per-lesion detection accuracy. The model correctly localized 680 of 724 annotated rib fractures (recall=94.0%) and generated 55 false-positive boxes. The mean average precision at an intersection-over-union threshold of ≥0.5 was 0.65, indicating robust lesion-level localization (Table 1).

**Table 1.** Dataset composition and performance of the artificial intelligence (AI) model using retrospective emergency department chest radiographs, including case-level detection and object-level localization (intersection-over-union [IoU]≥0.50).

Category and metric	Value
Dataset	
Total images	262
Ground-truth boxes	724
Case-level detection (%)	
Sensitivity (recall)	89.6
Precision	96.6
*F*_1_-score	0.93
Object-level localization	
Recall	94.0%
mAP^a^ (IoU≥0.5)	0.65

a mAP: mean average precision.

**Figure 2.**
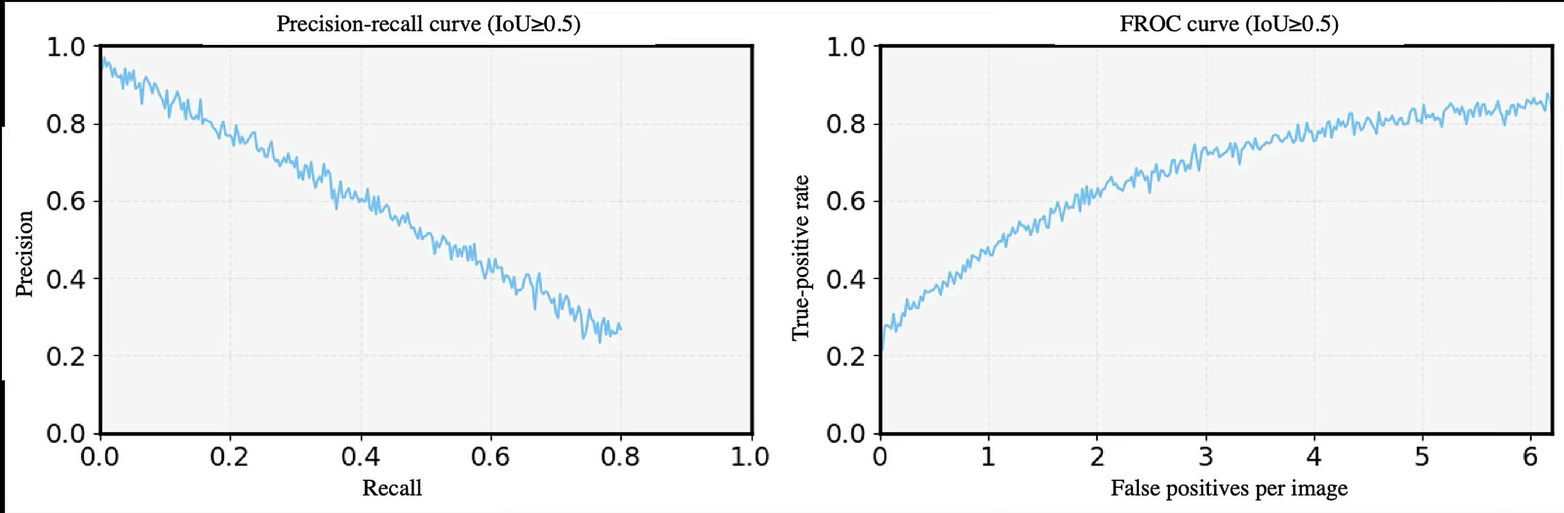
The performance of the artificial intelligence (AI)–assisted rib fracture detection model was evaluated in the retrospective model development and validation dataset using emergency department chest radiographs. (A) Precision-recall curve demonstrating case-level detection performance at an intersection-over-union (IoU) threshold of ≥0.50 (area under the curve=0.65). (B) Free-response receiver operating characteristic (FROC) curve showing lesion-level sensitivity as a function of false positives per image, with localization performance assessed at the same IoU threshold (mean average precision=0.65; recall=94%).

Curve-based analyses further characterized the model’s detection behavior (Figure 2). The precision-recall curve (Figure 2A) maintained precision ≥0.90 until recall fell below 0.55 (precision-recall area under the curve=0.65), demonstrating high reliability across a broad sensitivity range. The free-response receiver operating characteristic (FROC) curve (Figure 2B) showed true-positive rates of 0.77 at 1 false positive per image and 0.88 at three, representing practical trade-offs between sensitivity and alert frequency in potential clinical deployment.

### Prospective Evaluation of AI Model in Emergency Department Workflow

The trained model was prospectively evaluated in parallel with clinical workflow, performing automated inference on incoming emergency radiographs. During the automated inference process, all incoming radiographs were standardized and resized to a fixed resolution of 512×512 pixels. The prospective evaluation cohort (April-July 2023) was temporally and operationally independent from the retrospective training and validation dataset (2010-2020), and no patient overlap existed between the 2 cohorts. During the prospective evaluation phase, all chest and rib radiographs from emergency department encounters were automatically processed by the AI system without disrupting clinical workflows. The bounding box outputs were logged for research analysis but were not disclosed to radiologists or used for patient management.

### Performance Assessment Using NLP-Derived Labels

To evaluate AI performance in the real-world setting, output was compared to formal radiology reports issued by board-certified radiologists. A rule-based natural language processing (NLP) pipeline was developed to extract structured rib fracture labels (positive, negative, or ambiguous) from free-text reports. The algorithm combined keyword detection (eg, “rib fracture,” “fx”) and negation handling (eg, “no evidence of,” “no definite fracture”).

To validate NLP accuracy, a random sample of 200 radiology reports was manually reviewed by 2 emergency physicians blinded to both NLP and AI results. The NLP classification achieved 96.5% agreement (193/200) with manual review, with a Cohen κ of 0.91, indicating excellent concordance. Most discrepancies were due to ambiguous language or complex negation structures (Table 2).

**Table 2.** Confusion matrix comparing natural language processing (NLP)–extracted radiology report labels with manual expert review in a randomly selected subset of 200 emergency department chest radiographs, used to assess labeling accuracy in the retrospective dataset.

Manual review	Fracture present	Fracture absent	Ambiguous	Total (NLP prediction)
NLP: fracture present	95	2	1	98
NLP: fracture absent	3	90	2	95
NLP: ambiguous	1	2	4	7
Total (manual review)	99	94	7	200

Formal radiology reports served as the primary reference standard for AI evaluation. This approach may have underestimated AI sensitivity because CT confirmation was not performed systematically. In select discordant cases—where the AI flagged fractures not documented in the reports—subsequent CT scans confirmed some of these findings. These discrepancies were retrospectively reviewed to investigate potential underreporting by radiologists. While informative, these exploratory adjudications were not used as a universal reference standard due to inconsistent CT availability. Nonetheless, radiology reports remained the definitive benchmark for all performance metrics. Additionally, the selected misclassified cases were examined to identify recurring patterns of diagnostic oversight among frontline physicians.

### Targeted Adjudication of Discordant Cases

To further explore potential underreporting within the report-based reference standard, we performed a focused review of discordant cases in which the AI system flagged suspected rib fractures not documented in the corresponding formal radiology reports. Because not all discordant cases had confirmatory CT imaging, an illustrative subset of 11 cases was selected based on the availability of same-encounter chest CT and clinical relevance for qualitative adjudication. Each case was reviewed to determine whether the AI-predicted fractures corresponded to true fractures confirmed on CT. These adjudications were exploratory and intended to contextualize the potential clinical value of AI detection beyond the report-based benchmarking.

### Data Analysis

Case-level performance was assessed at the radiographic study (accession) level by comparing AI outputs to NLP-derived labels from radiology reports. A study was considered positive if at least 1 image within the same examination was flagged as having a rib fracture by the AI system; otherwise, the study was classified as negative. Key metrics included sensitivity, specificity, accuracy, positive predictive value, negative predictive value (NPV), and *F*_1_-score. Ninety-five percent CIs were calculated using nonparametric bootstrap resampling (1000 iterations). The results were summarized in confusion matrices and diagnostic performance plots.

### Statistical Analysis

All statistical analyses were performed using Python 3.11 (pandas v2.2, scikit-learn v1.4) and R 4.3.2. Continuous variables were reported as mean (SD) or median with IQR. Categorical variables were summarized as counts and percentages. A 2-tailed *P*<.001 was considered statistically significant.

This study was reported in accordance with the Checklist for Artificial Intelligence in Medical Imaging (CLAIM) guideline, with the completed checklist provided as Checklist 1.

## Results

### Study Cohort

From April 1 to July 2, 2023, all chest and dedicated rib radiographs acquired in the emergency department were automatically processed by the AI system in a parallel workflow, yielding 23,251 imaging studies from 20,908 unique patient visits. Population demographics are summarized in Table 3. The mean age was 55.9 years (SD 22.3; range 0‐106), with 10,770 (51.5%) male and 10,138 (48.5%) female patients. A radiologist review identified 589 rib-fracture cases (prevalence 2.8%).

**Table 3.** Demographic and clinical characteristics of patients in the emergency department included in a prospective observational study of artificial intelligence (AI)–assisted rib fracture detection (April 1-July 2, 2023).

Characteristic	Value
Total cases	20,908
Age (y), mean (SD; range)	55.9 (22.3; 0‐106)
Sex, n (%)	
Male	10,770 (51.5)
Female	10,138 (48.5)
Radiologist-confirmed rib fractures, n (%)	589 (2.8)

### AI Model Performance

AI model outputs were compared on a per-case basis against structured rib-fracture labels derived from board-certified radiology reports. At the selected operating point—corresponding to approximately one false-positive per image on the FROC curve—the system achieved a sensitivity of 0.745 (95% CI 0.708‐0.780) and specificity of 0.933 (95% CI 0.930‐0.937). Positive predictive value was 0.242 (95% CI 0.223‐0.262), and negative predictive value was 0.992 (95% CI 0.991‐0.994). The overall *F*_1_-score was 0.365 (95% CI 0.340‐0.390) with an accuracy of 0.928 (Table 4).

**Table 4.** Case-based diagnostic performance of an artificial intelligence–assisted rib fracture detection system in a prospective observational emergency department study^a^.

Metric	Estimate (95% CI)
Sensitivity	0.745 (0.708‐0.780)
Specificity	0.933 (0.930‐0.937)
PPV^b^	0.242 (0.223‐0.262)
NPV^c^	0.992 (0.991‐0.994)
*F*_1_-score	0.365 (0.340‐0.390)
Accuracy	0.928 (N/A^d^)

a Performance metrics are reported with 95% CIs using final radiologist reports as the reference standard.

b PPV: positive predictive value.

c NPV: negative predictive value.

d N/A: not available.

**Figure 3.**
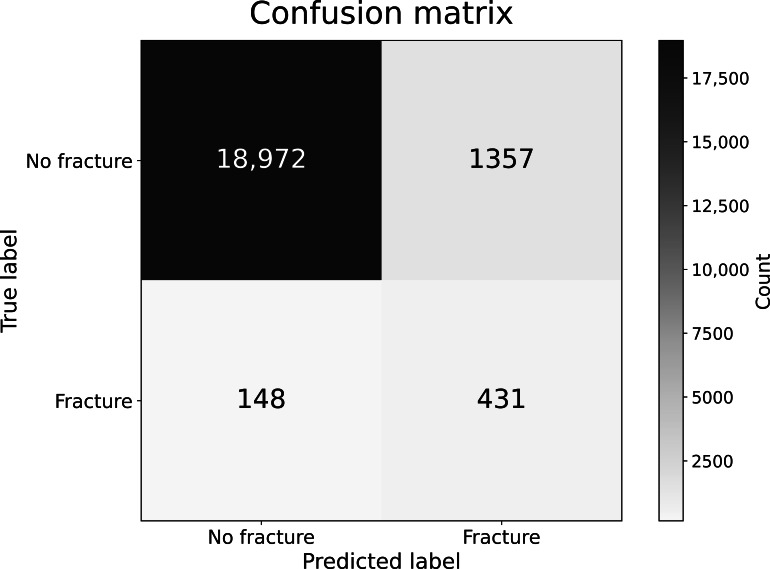
Case-level confusion matrix of the artificial intelligence (AI)–assisted rib fracture detection system during prospective emergency department deployment, using final radiologist reports as the reference standard. Darker blue indicates a higher number of cases (count), as shown in the color bar.

As shown in Figure 3, the AI system correctly identified 431 (74.5%) fracture-positive cases while producing 1357 (6.1%) false positives and 148 (0.7%) false negatives across 23,251 studies. This distribution demonstrates the model’s high true-negative count (n=18,972, 93.3%) and its strong negative predictive value during deployment in the emergency department. No temporal drift or learning curve effects were observed, as the deployed model remained fixed throughout the study period.

### Imaging Workload Patterns

**Figure 4.**
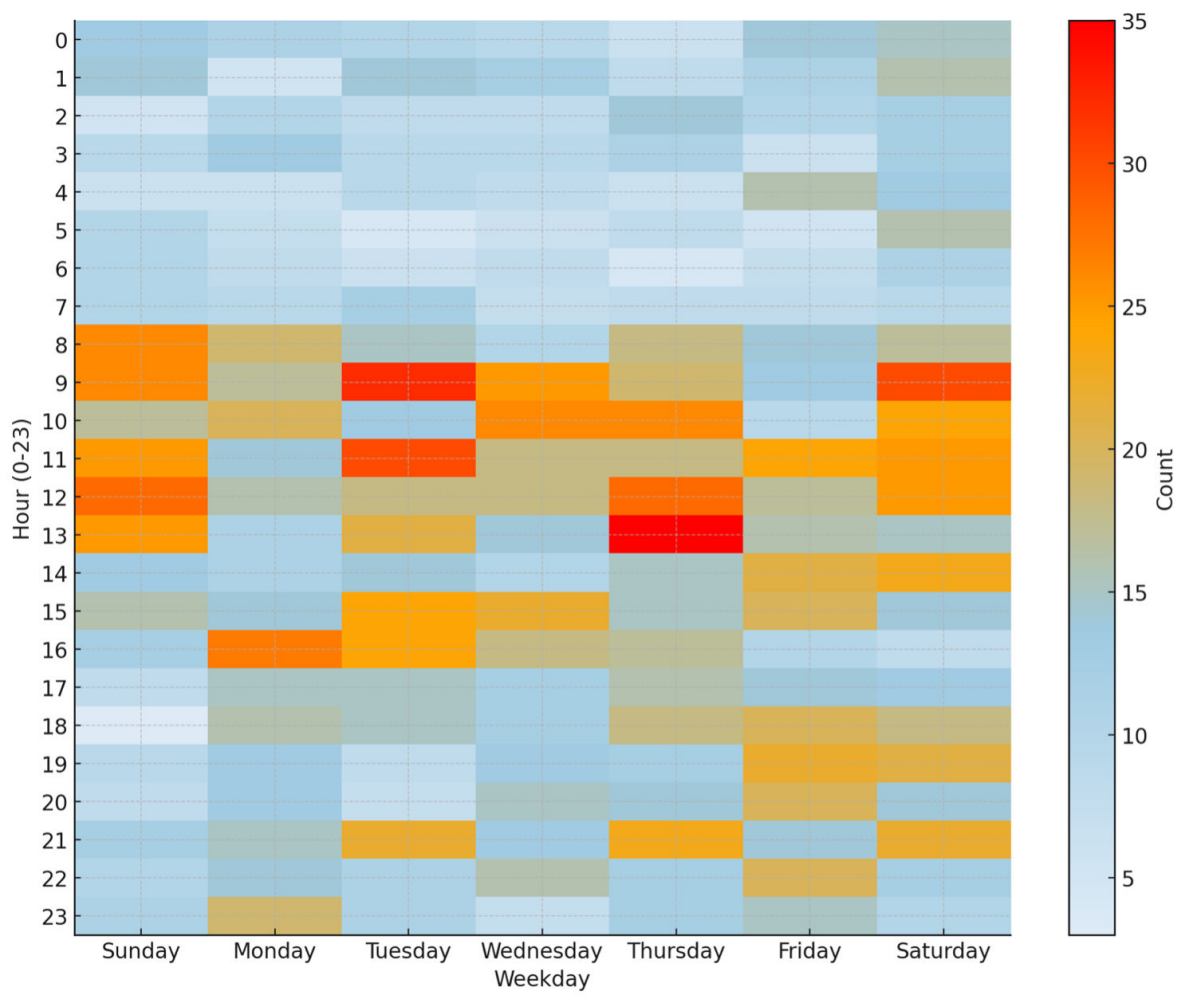
Heatmap illustrating the temporal distribution of chest radiograph imaging workload by hour of day and weekday during prospective emergency department deployment. Color intensity represents the number of chest radiographs acquired per hour, highlighting peak imaging periods across weekdays and weekends.

Analysis of imaging demand revealed predictable diurnal and weekly patterns, with peak volumes between 08:00 and 16:00 daily and secondary surges on Thursday to Saturday evenings. Demand was the lowest between 00:00 and 07:00 across all days of the week (Figure 4).

### Inference Turnaround Time

**Figure 5.**
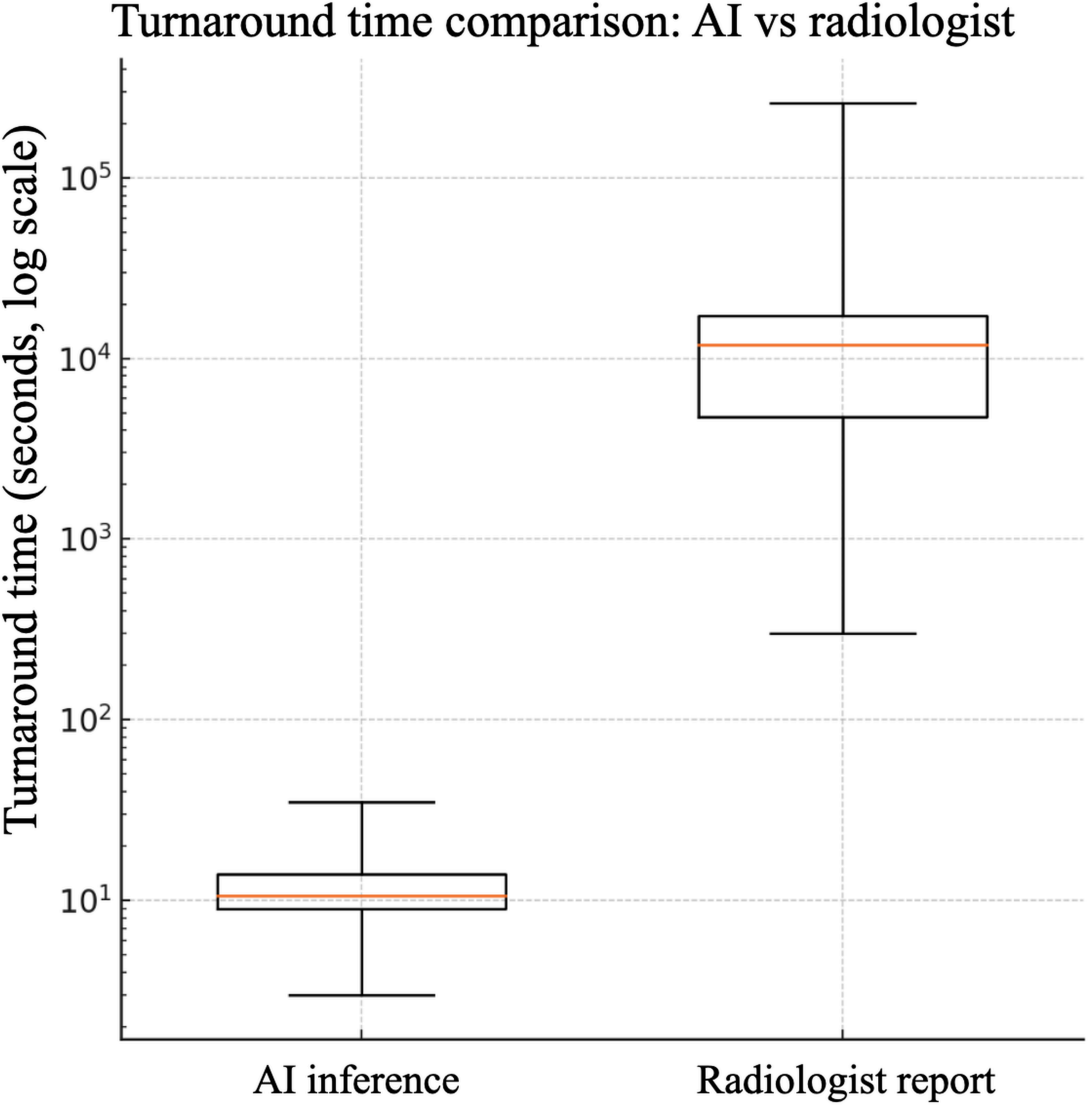
Comparison of turnaround times between artificial intelligence (AI) inference and radiologist reporting during prospective emergency department deployment (April 1-July 2, 2023). Boxplots on a logarithmic scale illustrate differences in processing time distributions between real-time AI inference and routine clinical reporting.

A total of 19,641 paired cases were included to compare AI inference and radiologist report turnaround times. As shown in Table 5, the AI system achieved a median processing time of 10.6 seconds per image (IQR 9.0‐14.0; range 3‐35 s), compared with a median of 3.3 hours (IQR 1.31‐4.80; range 0.08‐72 h) for radiologist reports. This represents a more than 1000-fold reduction in turnaround time. Figure 5 illustrates this disparity using boxplots on a logarithmic scale. A paired Wilcoxon signed-rank test confirmed that AI inference was significantly faster than radiologist reporting (*W*=112,987.5; *P*<.001). This median reporting time reflects the full clinical workflow, including overnight and backlog delays typical of high-volume emergency radiology settings.

**Table 5.** Turnaround times for artificial intelligence (AI) inference versus radiologist reporting during prospective emergency department deployment (n=19,641)^a^.

Metric	AI inference time (s)	Radiologist report time (s)
Mean	10.9	10,877
Standard deviation	3.0	4008
Median (50%)	10.6	11,880
IQR (25%‐75%)	9.0‐14.0	4728‐17,280
Min	3.0	300
Max	35.0	259,200

a Times are summarized using descriptive statistics, including median and interquartile range.

This processing speed highlights the potential of AI-assisted triage systems to complement radiology workflows by rapidly identifying cases for prioritized review, especially in high-volume emergency settings.

### System Reliability

During the prospective evaluation of the AI system operating in a parallel clinical workflow, the AI platform experienced 1 service interruption—from 27 April to 10 May 2023—caused by GPU overload that halted all inference operations. A total of 3610 studies acquired during this 14-day outage were excluded from the turnaround time analysis but retained in the diagnostic accuracy evaluation (their formal radiology reports remained available). Following hardware replacement, full functionality was restored on 11 May, and no further outages occurred over the remainder of the study period.

### Illustrative Review of Discordant Cases

To further evaluate the AI system’s potential diagnostic value beyond report-based benchmarking, a targeted adjudication was conducted on 11 representative discordant cases in which the AI system flagged suspected rib fractures not described in the corresponding radiology reports (Table 6). Among these, 7 cases (cases 1‐7) were subsequently confirmed as true fractures on CT (“AI-CT concordant”), indicating that several AI-labeled false positives in the report-based analysis represented true fractures missed in the reference standard. The remaining 4 cases (cases 8‐11) were confirmed negative on CT, primarily attributable to nonfracture anatomical structures or imaging artifacts. Including these CT-confirmed fractures as true positives would modestly increase the model’s effective sensitivity and positive predictive value, highlighting the underestimation inherent in report-based benchmarking.

**Table 6.** Targeted post hoc computed tomography (CT) adjudication of representative discordant cases in which artificial intelligence (AI) flagged suspected rib fractures not described in the corresponding radiology reports during prospective emergency department deployment.

Case	AI output	Radiologist report	Emergency physician	Outcome	Note (AI significance)
1	Flagged right rib fracture	No fracture	Noted with POCUS^a^	CT-confirmed	Triage value, prompting clinicians to perform US^b^
2	Flagged right rib fracture	No fracture	Missed	CT-confirmed	AI-CT concordance
3	Flagged left fifth rib fracture	No fracture	Missed	CT-confirmed	AI-CT concordance
4	Flagged rib fracture post chest tube	No fracture	Missed	CT-confirmed	Chest tube artifact did not impair detection
5	Flagged lower-rib fracture	No fracture	Missed	CT-confirmed	AI-CT concordance
6	Flagged fracture near hardware	No fracture	Missed	CT-confirmed	Detected fracture adjacent to surgical hardware
7	Flagged fracture under scapular shadow	No fracture	Missed	CT-confirmed	AI-CT concordance
8	Flagged fracture at scapula border	No fracture	No fracture	False positive	Scapular margin misidentified
9	Flagged rib fracture at bra clasp	No fracture	No fracture	False positive	Bra hardware artifact
10	Flagged fracture at chest tube marker	No fracture	No fracture	False positive	Chest tube marker misinterpreted
11	Flagged fracture (image noise)	No fracture	No fracture	False positive	Image noise

a POCUS: point-of-care ultrasound.

b US: ultrasound.

**Figure 6.**
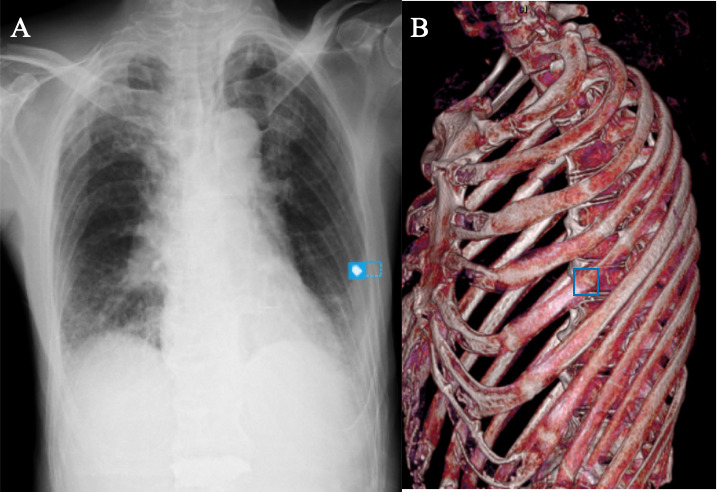
Representative true-positive rib fracture detected by artificial intelligence (AI) and confirmed by computed tomography (CT) during prospective emergency department deployment. (A) Chest radiograph showing an AI-flagged fracture of the left fifth rib that was not described in the initial radiology report. (B) Corresponding CT image confirming the fracture at the same anatomical location.

**Figure 7.**
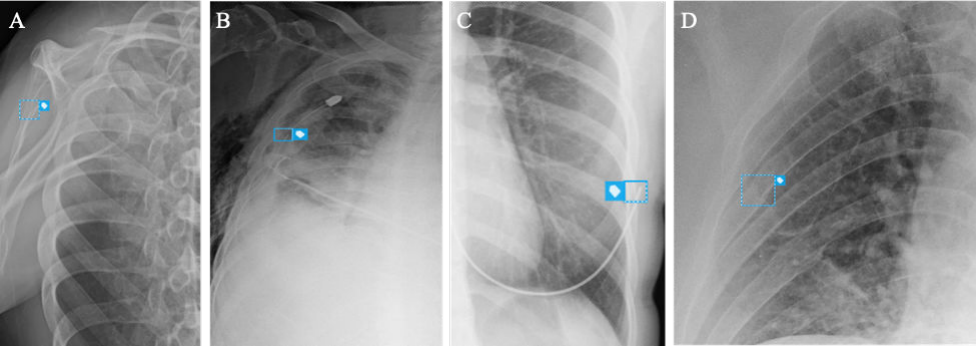
Representative false-positive detections generated by an artificial intelligence (AI)–assisted rib fracture detection system during prospective emergency department deployment. The examples illustrate common sources of false-positive signals on chest radiographs. (A) Scapular margin overlap misinterpreted as a rib fracture. (B) Chest tube marker misidentified as a rib discontinuity. (C) Bra hardware producing a linear opacity mimicking a fracture. (D) Image noise and low-contrast regions leading to spurious detection.

In one representative case (Case 3), the AI system correctly identified a subtle nondisplaced fracture of the left fifth rib that was not documented in the radiology report but later verified on 3D CT reconstruction (Figure 6). In contrast, Figure 7 illustrates the main sources of false positives, including scapular margin misinterpretation, chest-tube hardware artifacts, and motion-induced noise.

This targeted CT adjudication underscores the potential of AI-assisted screening to augment clinical vigilance by identifying subtle or overlooked fractures, while also emphasizing the need to improve artifact robustness and optimize false-positive suppression for practical clinical integration.

### Common Pitfalls in Frontline Rib Fracture Detection

Our review of discordant cases identified 3 principal drivers of missed rib fractures by emergency physicians: first, non-thoracic presenting complaints (eg, catheter malfunction or abdominal pain) led interpreters to focus on unrelated findings and overlook subtle rib breaks; second, the absence of classic chest pain—patients describing only mild discomfort or a vague “pop”—lowered clinical vigilance for nondisplaced fractures; and third, competing urgent injuries (facial, limb, or soft-tissue trauma) diverted attention from the chest, resulting in underappreciated fractures.

## Discussion

### Principal Findings

In this observational feasibility study using real-world emergency department imaging data, we demonstrated that a faster R-CNN–based AI system can operate in parallel with routine clinical workflows to provide near-instantaneous rib fracture triage without influencing patient care. During a 3-month evaluation period, the model automatically processed 23,251 CXRs with a median inference time of 10.6 seconds per image, achieving a turnaround time reduction exceeding 3 orders of magnitude compared with formal radiologist reporting, while maintaining 74.5% sensitivity and 93.3% specificity. These findings position AI as a potential automated screening aid capable of rapidly identifying low-risk examinations and generating signals that could inform future prioritization strategies. The observed discrepancy between the 10.6-second AI inference time and the 3.3-hour radiologist turnaround time reflects a critical clinical bottleneck in busy emergency departments. Although these metrics represent different stages—technical processing versus final clinical documentation—the delay in official reporting highlights the diagnostic gap AI aims to address. In this context, near-instantaneous AI alerts may support case prioritization before formal reporting, although no clinician-facing alerts were implemented in this study.

However, the current findings primarily demonstrate technical feasibility rather than full clinical feasibility, as the AI system operated passively without direct clinician interaction. Future clinician-in-the-loop evaluations will be necessary to assess workflow integration, usability, and impact on diagnostic behavior or patient outcomes.

Although the system’s positive predictive value was relatively low (24.2%), this trade-off aligns with its design as a triage support tool rather than a stand-alone diagnostic system. In high-volume emergency departments, the ability to rapidly identify examinations with a low likelihood of fracture is crucial. The model’s high NPV of 99.2% allows clinicians to focus on a smaller, higher-risk subset of cases, thereby improving efficiency and reducing cognitive load. Given the observed sensitivity of 74.5%, false-negative cases remain possible, and the system should not be used as a stand-alone rule-out mechanism or as a substitute for clinical judgment.

### Comparison to Prior Work

Similar findings have been reported by Yao et al [20], who demonstrated that deep learning systems with high NPV on chest CT can reduce radiologists’ workload by effectively identifying nonfracture cases. A recent systematic review by van den Broek et al [21] further emphasized the triage value of AI in fracture detection, underscoring its potential across multiple imaging modalities.

Our AI system also demonstrated robust performance across circadian and weekly imaging surges, with peak volumes observed between 08:00 and 16:00 and during Thursday to Saturday evenings. However, a 14-day GPU hardware outage during the study period highlighted a real-world challenge of maintaining the AI system’s reliability in clinical environments. This incident underscores the need for infrastructure redundancy, real-time monitoring, and failover protocols—key considerations for sustainable AI deployment. These practical aspects of AI deployment remain underreported in most published studies [22].

Compared with prior approaches such as the PACS-AI platform [23], our system offered full automation, operating continuously in real time and without the need for manual image selection. This better reflects the demands of frontline emergency radiology. Herpe et al [24] demonstrated improved diagnostic accuracy with PACS-integrated AI for limb fractures; however, their study did not evaluate scalability or autonomous triage capability under high-throughput conditions. In contrast, our study incorporated prospective data collection within a real-world emergency department workflow, allowing the assessment of AI performance, reliability, and operational feasibility under authentic clinical conditions.

Focused adjudication of discordant cases revealed that the AI system correctly identified rib fractures that were missed by both radiologists and emergency physicians in 7 cases, all subsequently confirmed on follow-up CT (“AI-CT concordant”). These findings highlight the potential of AI to strengthen diagnostic vigilance in complex clinical scenarios. While prior studies—such as Zhou et al [25]—have shown that AI can detect rib fractures overlooked in initial CT interpretations, with confirmation on follow-up imaging, these investigations have largely centered on CT-based workflows. Although CT is highly sensitive, its routine use is limited by concerns over radiation, cost, and logistics. In contrast, our CXR-focused approach targets the most widely used imaging modality in acute care, offering a more scalable and practical solution for real-world emergency triage. Notably, Brady et al [26] have emphasized that diagnostic errors and discrepancies are not uncommon in radiology, with daily error rates estimated at 3%‐5%, reinforcing the importance of AI as a complementary tool to enhance diagnostic accuracy.

Four false-positive cases revealed predictable pitfalls, including the misinterpretation of scapular margins, chest-tube hardware, and motion artifacts. Similar findings were reported by Sun et al [27], who noted frequent false positives in an AI model for rib fracture detection on CXR, often due to anatomical overlap and imaging artifacts. These results support the need for artifact-aware retraining and preprocessing optimization to reduce false alerts and improve clinical integration.

This targeted CT adjudication further highlights the complementary role of AI in identifying subtle or overlooked fractures and underscores the inherent limitation of using report-based labels as the reference standard in real-world studies.

### Future Directions

In addition, integrating AI-generated alerts into emergency radiology workflows will require careful calibration of alert thresholds to minimize false positives and prevent alert fatigue among clinicians. Human-centered design, interface refinement, and iterative feedback from end users will be critical to achieving effective and sustainable adoption.

While most prior prospective studies have emphasized diagnostic performance or radiologist feedback, our findings extend beyond these metrics to include diagnostic efficacy, operational resilience, and system failure contingencies. These real-world insights support the feasibility and clinical value of embedding AI into routine emergency department workflows. Recent work has highlighted the importance of not only measuring accuracy but also assessing robustness across patient and workflow variability [28]. Furthermore, the need for deployment frameworks that address hardware resilience, continuous quality monitoring, and interpretability safeguards is increasingly recognized as essential for sustainable AI adoption in high-acuity settings [29].

Recent reports and position statements have highlighted a persistent gap between the promising diagnostic performance of AI systems and their limited demonstrated clinical benefit. Robust, prospective, and randomized clinical studies remain urgently needed to justify large-scale implementation [30,31]. Even high-performing AI models (area under the curve≈0.85) have failed to surpass standard clinical practice in improving patient outcomes [32,33]. These findings reinforce ongoing concerns that most AI or machine learning devices, despite regulatory authorization, are primarily validated using retrospective data and therefore remain susceptible to selection bias, distributional shift, and overestimation of generalizability [34,35].

Clinical decision-making in emergency care is inherently multimodal: physicians integrate imaging findings with the mechanism of injury, examination, and vital signs to guide judgment. In contrast, this AI system analyzes images in isolation and is designed not to replace but to support clinicians as a rapid screening aid—enhancing vigilance in high-volume, high-pressure environments where missed fractures may occur. Incorporating multimodal clinical data in future models could further improve diagnostic relevance and workflow integration.

Although this was an observational feasibility study, it represents one of the largest evaluations of an AI-assisted rib fracture detection system in real-world emergency radiology. The findings demonstrate that such a system can provide meaningful diagnostic support, maintain consistent performance at scale, and potentially enhance patient safety. Future implementation should therefore shift from technical to clinical feasibility, focusing on clinician-in-the-loop impact studies, PACS-integrated trials, and workflow efficiency assessments. Although user perception was not formally assessed, informal feedback from emergency physicians indicated strong interest in AI-supported flagging—particularly for subtle fractures and during periods of high patient volume.

Future research should prioritize prospective, multicenter studies to validate generalizability and quantify AI impact on workflow, resource utilization, and patient outcomes. Model improvements—including artifact-aware retraining, expanded fracture coverage in challenging scenarios such as subtle or anatomically obscured fractures, and continuous learning—will be critical to enhance diagnostic precision. Finally, building infrastructure resilience and integrating effective alert management into radiologist workflows are essential for sustainable clinical adoption.

### Limitations

First, this single-center observational study may limit generalizability to other institutions with different imaging protocols, patient populations, or workflow environments. Second, during retrospective model development, training and validation were performed at the image level rather than the patient level. This may have resulted in optimistic internal validation estimates due to potential within-patient similarity, which likely contributes to the observed performance gap between retrospective validation and prospective real-world deployment. Additionally, stratified performance analyses by age group and sex were not performed due to the low prevalence of rib fractures in certain subgroups, particularly pediatric patients. Similarly, the small fraction of oblique views (approximately 1.3%) prevented a dedicated analysis by imaging view, as the limited sample size would yield unstable estimates for these specific cohorts.

The prospective evaluation period also did not include winter months. Seasonal variation in trauma mechanisms or imaging artifacts may influence fracture detectability in certain settings; therefore, caution is warranted when generalizing these findings across different seasonal contexts.

Additionally, the use of a 512×512 resolution for model inference represents a technical trade-off; while it facilitates rapid processing, the associated downsampling may limit the detection of very subtle cortical disruptions.

Third, using radiology reports as the reference standard—while pragmatic—may underestimate the AI system’s true performance, as subtle or occult fractures can be underreported in clinical practice. A focused CT review of representative discordant cases further supported this concern, revealing instances where AI-predicted fractures were subsequently confirmed as true fractures on CT. This approach likely yielded conservative performance estimates, since NLP-derived labels may not capture subtle fractures identified by AI or CT.

Regarding the study design, although post hoc adjudication is generally more appropriate for hypothesis generation than for definitive performance reassessment, modifying the reference standard after study completion may introduce bias. Accordingly, in this study, performance evaluation was anchored to the contemporaneous clinical reference standard used in routine practice. More comprehensive adjudication strategies—such as consensus radiologist review of AI-positive, report-negative cases—may provide additional insights when implemented within a separately designed study.

Finally, because AI predictions were not disclosed to clinicians, we did not assess downstream clinical outcomes, including changes in diagnostic behavior, time to intervention, or patient management. As for the data pipeline, although we used NLP to extract rib fracture labels from radiology reports, which may introduce misclassification in ambiguous cases, the pipeline demonstrated high agreement with manual review (κ=0.91). Given the observed 3.5% discrepancy rate, any residual label noise propagating through the large-scale dataset may introduce modest uncertainty into performance estimates. However, the substantial sample size (n=23,251) is expected to attenuate the impact of such noise, supporting the stability of the resulting confidence intervals for large-scale clinical benchmarking.

### Conclusions

In this observational feasibility study, we evaluated a faster R-CNN–based AI system deployed in parallel with clinical workflows to automatically detect rib fractures on CXRs using real-world emergency department data. Although AI outputs were not visible to clinicians, the system processed over 23,000 studies with high throughput, achieving 74.5% sensitivity and 93.3% specificity and delivering results within seconds—over 1000 times faster than formal radiologist reports.

These findings demonstrate strong technical feasibility of real-time AI-assisted rib fracture detection in emergency radiology. While clinical decisions remained unaffected during this observational phase, future studies should validate clinical feasibility through clinician-in-the-loop evaluation, PACS integration, and workflow optimization to address potential alert fatigue and false-positive management.

## Supplementary material

10.2196/77965 Checklist 1 CLAIM (Checklist for Artificial Intelligence in Medical Imaging).

## Abbreviations

AI: artificial intelligence
AP: anteroposterior
CLAIM: Checklist for Artificial Intelligence in Medical Imaging
CT: computed tomography
CXR: chest radiograph
FROC: free-response receiver operating characteristic
GPU: graphics processing unit
NLP: natural language processing
NPV: negative predictive value
PACS: picture archiving and communication system
R-CNN: region-based convolutional neural network
