# Supplementary material for: Prospective Diagnostic Accuracy and Technical Feasibility of Artificial Intelligence-Assisted Rib Fracture Detection on Chest Radiographs: Observational Study
Source: JMIR Med Inform. 2026 Jan 29;14:e77965. doi: 10.2196/77965 (PMC12854400; doi:10.2196/77965)
Supplement: Checklist 1 [file medinform-v14-e77965-s001.pdf]

Multimedia Appendix 1. CLAIM (Checklist for Artificial Intelligence in Medical Imaging) Compliance

This supplementary document provides the completed CLAIM checklist, outlining the reporting compliance of this study with the Checklist for Artificial Intelligence in Medical Imaging (CLAIM) guideline.

Section	Item	Description	Reported (Yes/No)	Location in Manuscript
Title/Abstract	1	Clearly identifies the study as artificial intelligence or machine learning in medical imaging	Yes	Title, Abstract
Introduction	2	States the clinical motivation and relevance for using AI	Yes	Introduction
Methods - Data	3	Describes data source, inclusion/exclusion criteria, and preprocessing	Yes	Materials and Methods - Study Design
Methods - Ground Truth	4	Explains the reference standard and how labels were established	Yes	Materials and Methods - Performance Assessment Using NLP-Derived Labels
Methods - Model	5	Details the model architecture, training procedure, and hyperparameters	Yes	Materials and Methods - AI Model Development
Methods - Evaluation	6	Defines evaluation metrics and validation strategies	Yes	Materials and Methods - Model Validation, Data Analysis
Methods - Statistical Analysis	7	Describes statistical tests and confidence interval estimation	Yes	Materials and Methods - Statistical Analysis
Results	8	Reports model performance with confidence intervals	Yes	Results - Model Performance, Table 1
Results	9	Reports comparison with reference standard	Yes	Results - Real-world Evaluation, Figure 4
Results	10	Reports analysis of errors and failure modes	Yes	Results - Illustrative Review of Discordant Cases, Discussion
Discussion	11	Interprets findings and discusses limitations, generalizability, and clinical implications	Yes	Discussion
Reproducibility	12	Provides information about model reproducibility or availability of code/data	No	Not applicable (proprietary hospital AI system)
Reporting Standard	13	States adherence to CLAIM guideline	Yes	Materials and Methods - Statistical Analysis
